# A New Spectrophotometric Method for Determination of Phenylpropanolamine HCl in its Pharmaceutical Formulations via Reaction with 2,3,5,6-tetrachloro-1,4-benzoquinone

**Published:** 2010-06

**Authors:** M. I. Walash, N. El-Enany, S. Saad

**Affiliations:** *Department of Analytical Chemistry, Faculty of Pharmacy, University of Mansoura, Mansoura, Egypt*

**Keywords:** spectrophotometry, method for determination of Phenylpropanolamine HCl, 2,3,5,6-tetrachloro-1,4-benzoquinone, dosage forms

## Abstract

A selective and simple spectrophotometric method has been developed for the determination of phenylpropanolamine HCl (PPA) in its dosage forms. The method was based on the formation of a colored N-vinyl chlorobenzoquinone derivative of PPA through its reaction with 2,3,5,6-tetrachloro-1,4-benzoquinone in presence of acetaldehyde. The colored product exhibits maximum absorbance at 650 nm. Different experimental parameters affecting formation and stability of the product were carefully studied and optimized. The stoichiometry of the reaction was determined, and the reaction pathway was postulated. The absorbance concentration plot was rectilinear over the range of 5-100 μg/mL with Limit of Detection (LOD) and Limit of Quantitation (LOQ) of 0.244 μg/mL and 0.74 μg/mL respectively. The analytical performance of the method was fully validated, and the results were satisfactory. The proposed method was successfully applied to the determination of PPA in its commercial dosage forms including tablets, capsules and syrups with good recoveries. Statistical comparison of the results with those of the comparison method showed good agreement and proved that there was no significant difference in the accuracy and precision between the reference and the proposed methods. The mechanism of the reaction pathway was postulated.

## INTRODUCTION

Phenylpropanolamine is a largely indirect acting sympathomimetic with an action similar to ephedrine, it is orally administered for the treatment of nasal congestion. It is frequently used in mixture preparations for the relief of cough and cold symptoms. Other uses of phenylpropanolamine include the control of the urinary incontinence in some patients. It has also been used to suppress appetite in the management of obesity (1).

**Figure 1:**
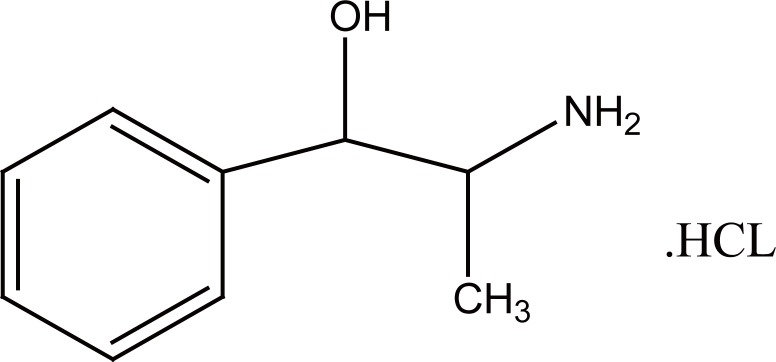
Structural formula of phenylpropanolamine. HCl (PPA).

PPA is Benzemethanol, α-(1-aminoethyl)-hydrochloride, (R^*^,S^*^)-, (±).(±) Norephedrine hydrochloride (Fig. 1). The USP (2) and the BP (3) recommended non aqueous titrimetric method for the determination of PPA in the pure form by adding mercuric acetate and titration with perchloric acid and using crystal violet as indicator, while both USP (2) and BP (3) recommended HPLC dertermination of PPA in dosage forms with UV detection at 210 nm. Due to its clinical advantages, PPA received a great interest. Several analytical techniques have been reported for PPA determination such as; titrimetry (4, 5), spectrophotometry (6-8), flourimetry (9), Raman spectroscopy (10), NMR (11), HPLC (2-15), CE (16-20), flow injection (21, 22), HPTLC (23), micellar chromatography (24-27), ion pair chromatography (28) and GC (29).

Concerning the published spectrophotometric method (6) it could be applied over the concentration range of 0.36-0.88 mg/mL, therefore it is less sensitive than the present sudy.

On the other hand, the other published spectrophotometric method (7) is depending on using the second derivative technique for the determination of PPA which is highly susceptible to interference from common tablets excipients.

The main advantage of the present study is the formation of colored product at 650 nm where interference from common tablet excipients are highly eliminated.

Spectrophotometry is considered the most convenient analytical technique, because of its inherent simplicity, low cost, and wide availability in most laboratories. The proposed method was based on the formation of a colored N-vinyl chlorobenzoquinone derivative of PPA by its reaction with 2,3,5,6-tetra chlorobenzoquinone (TCBQ) in the presence of acetaldehyde (ACD).

## EXPERIMENTAL

### Instruments

A shimadzu UV-Visible 1601 PC spectrophotometer (Kyoto, Japan) was used for spectrophotometric measurements (P/N 206-67001). The recording range was 0-1.2.

### Reagents and materials

All the reagents used were of Analytical Grade and distilled water is used throughout the work.

Phenylpropanolamine HCl with a purity of 99.89% (6) (Batch # 41204) was kindly supplied by EPICO (10^th^ of Ramadan Egypt).

- 2,3,5,6-tetrachloro-1,4-benzoquinone (TCBQ), (BDH Co. Ltd., England) was freshly prepared as 3 × 10^-2^ M prepared in 1,4-dioxan.
- 1,4-dioxan was obtained from (BDH. UK).
- Acetaldehyde (ACD) was obtained from Sigma Aldrich, Germany and was prepared 8% (v/v) in propane -2-ol.
- Chloroform, concentrated hydrochloric acid and sodium hydroxide were purchased from (BDH.UK).
- The following dosage forms containing the drug were purchased from local pharmacy.
- Flustop^®^ tablet, Batch # 043663A, each tablet labeled to contain 24 mg PPA, 3 mg chlorpheniramine maleate, 400 mg paracetamol and 32 mg caffeine, product of GlaxoSmithkline S.A.E, Elsalam city, Cairo, A.R.E.
- Contac 12^®^ capsule: Batch # 061682 each capsule labeled to contain 50 mg PPA and 4 mg of isopropamide, product of EPICO. (10^th^ of Ramadan Egypt).
- Pararhinol^®^ syrup (Batch # 431046), each 5 mL labeled to contain 6.25 mg PPA, 1mg chlorpheniramine maleate and 150 mg paracetamol, product of Misr Co. for pharmaceutical industry S.A.E.

### Preparation of the sample

An accurately weighed amount (100 mg) of PPA was dissolved in 10 mL distilled water. The solution was transferred quantitively to 100 mL separating funnel, and the solution was rendered alkaline with 5 mL of 10% NaOH solution. The liberated base was extracted with three times, each with 25 mL of chloroform. The combined organic extract was passed through anhydrous sodium sulphate into 100 mL volumetric flask, the volume was completed with chloroform to obtain a standard solution of 1000 μg/mL, calculated as the hydrochloride salt. This solution was further diluted with the same solvent to obtain the working solution. The solutions were found to be stable for at least one week when kept in the refrigerator.

### General recommended procedures

**Construction of calibration graph.** Aliquot volumes of the drug covering the working concentration range (5-100 μg/mL, final concentration) were quantitatively transferred to a series of 10 mL volumetric flask To each flask 1.2 mL of ACD solution (8% v/v in propan-2-ol) and 1.2 mL of TCBQ (3 × 10^-2^ M in 1,4-dioxan) were added. The solution was allowed to stand for 20 minutes at room temperature and the solution was diluted to the volume with propan-2-ol. The absorbance of the reaction product was measured at 650 nm *versus* a reagent blank prepared simultaneously. The calibration graph was constructed by plotting the absorbance *versus* the final concentration of the drug (μg/mL). Alternatively, the corresponding regression equation was derived.

**Assay procedure for tablets.** Ten tablets were weighed, pulverized and mixed well. A weighed quantity of the powdered tablets equivalent to 100 mg PPA was dissolved in 10 mL distilled water, sonicated for 15 minutes. The solution was firstly filtered then transferred quantitively to 100 mL separating funnel and the procedures described under "Construction of calibration graph" were performed. The nominal content of the tablets was calculated using the corresponding regression equation.

**Assay procedure for capsules and syrups.** The weighed quantity of the mixed and powedered contents of ten capsules or an accurately measured volume of the syrup equivalent to 100 mg of the drug were transferred to a small conical flask and was dissolved in 10 mL of distilled water. The solution was sonicated for 15 minutes, filtered and completed as in raw material.

## RESULTS AND DISCUSSION

### Results

Phenylpropanolamine is aweakly absorbing compound and it exhibits weak absorbance at λ_max_ 261 nm such problem is highly aggravated when it is necessary to determine the drug in pharmaceutical preparations.

**Figure 2:**
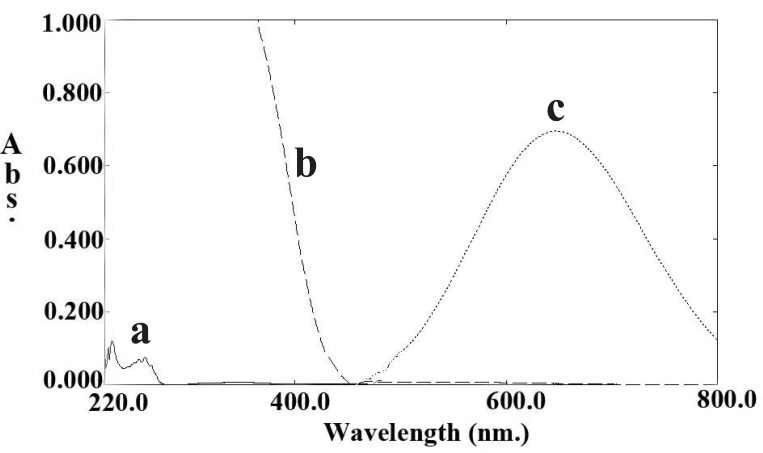
Absorption spectra of the reaction: (a) PPA (70 μg/mL) in chloroform; (b) Reagent blank; (c) Reaction product of (70 μg/mL) PPA with TCBQ.

Enamine formation is of particular interest in pharmaceutical analysis as it can be used as a basis for the quantitative analysis of many pharmaceutical compounds. Enamine is formed through the interaction of the amino group of the drug molecule with ACD with the formation of N-alkylamine (enamine). The enamine condenses with the TCBQ to give a highly colored vinylamino-subistituted quinone, which can be measured spectrophotometrically with a maximum absorbance at 650 nm. Several compounds of pharmaceutical interest were determined through such approach (30-32) (Fig. 2).

**Study of Experimental Parameters.** Different factors affecting color development and its stability were carefully studied and optimized. Each was changed while the others were kept constant

**Figure 3:**
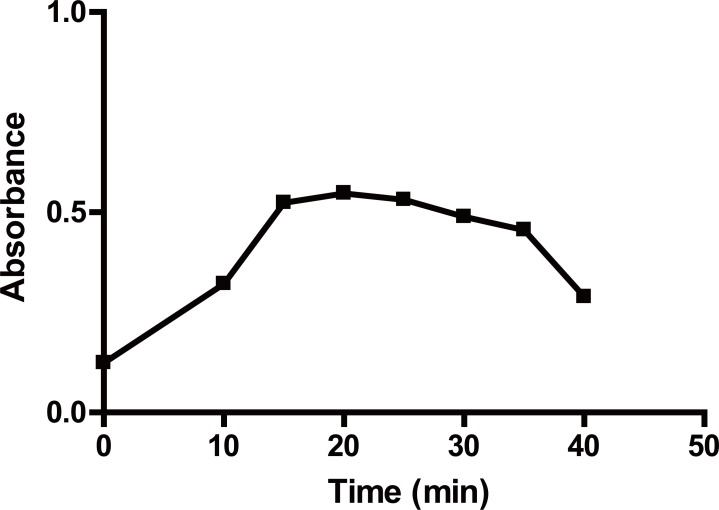
Effect of reaction time on the absorbance value of the reaction product of 100 μg/mL PPA with ACD 8%, v/v and TCBQ 3 × 10^-2^ M at room temperature.

**Figure 4:**
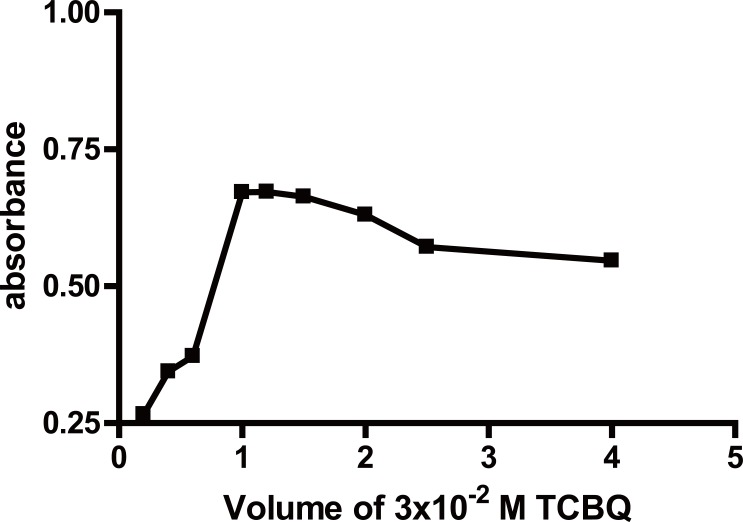
Effect of TCBQ on the absorbance values of the formed vinylamino subistituted haloquinone using 100 μg/mL of PPA.

**Figure 5:**
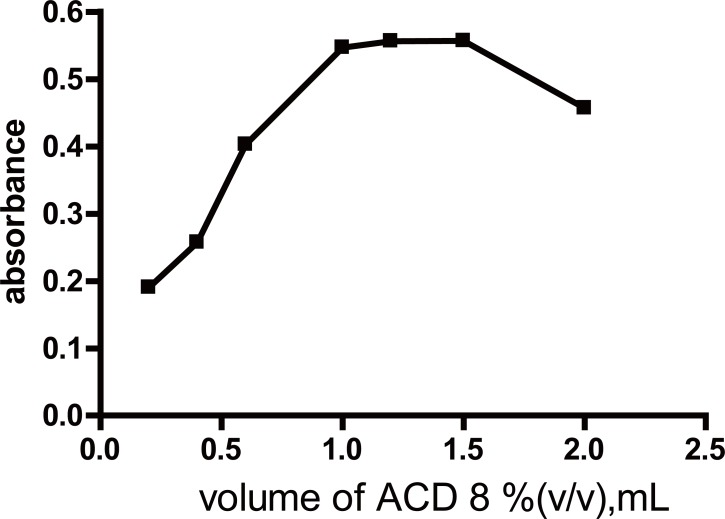
Effect of [ACD] volume on the absorbance value of the formed vinylamino subistituted halo quinone using 100 μg/mL PPA using 3 × 10^-2^ M TCBQ.

- **Effect of temperature and time.** The color intensity was found to have the maximum intensity at room temperature (25 ± 5)°C for 20 minutes (Fig. 3). Increasing time resulted in a slight decrease in the formed colored product. Upon increasing the temperature, the absorbance of the reaction product decreased gradually. Therefore the reaction was carried out at room temperature (Table 1).
- **Effect of the concentration of TCBQ solution.** The influence of the concentration of TCBQ was studied using different volumes of a 3 × 10^-2^ M solution of the reagent. It was found that increasing volumes of the reagent produces a proportional increase in the absorbance value of the reaction product up to 1 mL. However, no further increase in the absorbance value was observed upon increasing the volume of the reagent up to 1.5 mL, after which further increase produced a gradual decrease in the absorbance value. Therefore, 1.2 ± 0.2 mL of 3 × 10^-2^ M of TCBQ solution was chosen as the optimal volume of the reagent (Fig. 4) (Table 1).
- **Effect of ACD concentration.** The influence of the concentration of ACD was studied using different volumes of 8% (v/v) ACD solution of the reagent. It was found that increasing volumes of ACD resulted in proportional increase in the absorbance value of the reaction product up to 1.2 mL. However, no further increase in the absorbance value was observed upon increasing the volume of the reagent up to 1.5 mL, after which further increase produced a gradual decrease in the absorbance value. Therefore, 1.2 ± 0.2 mL of 8% (v/v) of ACD solution was chosen as the optimal volume. (Fig. 5) (Table 1)
- **Effect of diluting solvent.** The effect of diluting solvent was tested using different solvents *viz methanol*, propan-2-ol, dioxan, toluene, benzene, dimethylformamide and propanol. Using propan-2-ol as diluting solvent gives highest absorbance value. Of all the solvents studied, the highest absorbance value with maximum product stability was obtained upon using propan-2-ol.
- **Effect of time on the stability of the formed adduct.** The reaction product was found to be stable at room temperature for approximately 70 minutes after which the absorbance value of the reaction product very slightly decreased.

**Table 1:** Analytical parameters for the determination of PPA by the proposed method

Parameter	Proposed method

Standard conc. (μg/mL)	1000
TCBQ conc.	3 × 10^-2^ M
TCBQ volume (mL)	1.2 ± 0.2
ACD conc (v/v)	8%
ACD volume (mL)	1.2 ± 0.2
Temperature (°C)	25 ± 5
Time (min)	20 ± 5
Stability of the product (min)	70
Suitable diluting solvent	Propan-2-ol
λ_max_ (nm)	650

### Validation of the proposed Methods

The validity of the proposed methods was tested regarding linearity, specificity, accuracy, repeatability and precision according to ICH Q2 (R1) recommendations (33).

**Linearity.** The calibration graphs obtained by plotting the values of the absorbance *versus* the final concentrations were found to be rectilinear over the concentration ranges cited in (Table 2). The proposed method was evaluated for the accuracy as percent relative error (% Er) and the precision as percent relative standard deviation (% RSD) (Table 2).

**Table 2:** Performance data for the proposed methods

Parameter	Method

Concentration range (μg/mL)	5-100
Limit of detection (LOD) (μg/ml)	0.24
Limit of quantification (LOQ) (μg/mL)	0.74
Correlation coefficient (r)	0.9999
Slope	0.0111
Intercept	0.021
S_y/x_	3.01 × 10^-3^
S_a_	8.21 × 10^-4^
S_b_	3.21 × 10^-5^
%RSD	0.3346
% Error	0.11
ε (l mol^-1^ cm^-1^.)	2180.73

S_y/x_, Standard deviation of the residuals; S_b_, Standard deviation of the slope; % Error, % RSD/√n; S_a_, Standard deviation of the intercept; ε, Molar absorptivity.

**Limit of quantitation and limit of detection.** The limits of quantitation (LOQ) was calculated according to ICH Q2B (R1) recommendation (33). The results are shown in (Table 2). The limit of detection (LOD) was calculated according to ICHQ2 (R1). The results are also summarized in (Table 2).

LOQ and LOD were calculated according to the following equations (33):

LOQ=10 S_a_/b

LOD=3.3 S_a_/b

where S_a_ is the standard deviation of the intercept of regression line, and b is the slopeof the calibration curve.

**Accuracy.** To test the validity of the proposed method it was applied for the determination of pure sample of PPA over the concentration range cited in (Table 1). The results obtained were in good agreement with those obtained using the comparison method. Statistical analysis of the results obtained using Student’s t-test and the variance ratio F-test (34) revealed no significance differences between the performance of the proposed and comparison methods regarding the accuracy and precision, respectively (Table 3). The spectrophotometric comparison method (35) based on determination of the studied drug through its reaction with 1, 2- naphthoquinone -4- sulphonate.

**Table 3:** Application of the proposed and comparison methods for the determination of PPA in pure form

Parameter	Proposed method			Comparison method (35)
	Conc. taken (μg/mL)	Conc. found (μg/mL)	% Found	

	5.00	4.98	99.63	
	10.00	9.98	99.82	
	20.00	19.98	99.90	
	30.00	29.98	99.93	
	40.00	39.98	99.95	99.53
	50.00	49.98	99.96	102.01
	60.00	59.98	99.96	100.11
	70.00	69.23	98.90	
	80.00	79.98	99.98	100.55 ± 1.30
	100.0	99.98	99.98	
X ± S.D.	99.8 ± 0.33			
t	0.19 (2.20)^a^			
F	1.54 (19.38)^a^			

Each result is the average of three separate determinations.

a Values between parenthesis are the tabulated t and F values respectively, at *p*=0.05 (34).

The validity of the methods were proved by statistical evaluation of the regression line, using the standard deviation of the residuals (*S_y/x_*), the standard deviation of the intercept (*S_a_*) and standard deviation of the slope (*S_b_*). The results are abridged in (Table 2). The small values of the figures indicate low scattering of the calibration points around the calibration line and high precision.

### Statistical Analysis

**Precision.**

- **Intraday precision.** The repeatability was tested by applying the proposed method for the determination of three concentrations of PPA in pure form (10, 20 and 80 μg/ mL) on three successive times. The mean % recoveries was 101.40% ± 0.99 with % relative standard deviation of 0.98 and % error of 0.56 respectively. Thus, indicates high accuracy and high precision of the proposed method. The results are presented in (Table 4).
- **Intermediate precision.** Intermediate precision was tested by repeated analysis of PPA in pure form using the concentrations shown in (Table 4) for a period of 3 successive days.

**Table 4:** Validation of the proposed methods for the determination of PPA in pure form

Parameter	Intra-day precision			Inter-day precision		
	Conc. taken (μg/mL)	Conc. found (μg/mL)	% Found	Conc. taken (μg/mL)	Conc. found (μg/mL)	% Found

	10.00	10.20	102.10	10.00	10.14	101.4
	20.00	19.64	98.25	20.00	19.80	99.0
	80.00	80.6	100.7	80.00	80.09	100.11
X± S.D.		101.4 ± 0.99			100.17 ± 1.2	
% RSD			0.98			1.19
% Error			0.56			0.69

Each result is the average of three separate determinations.

The mean % recoveries was 100.17% ± 1.2 with % relative standard deviation of 1.19 and % error of 0.69 respectively. Thus, indicates high accuracy and high precision of the proposed method.

**Robustness of the method.** The robustness of the method adopted is demonstrated by the constancy of the absorbance with the deliberated minor changes in the experimental parameters such as the change in the volume of TCBQ 3 × 10^-2^ M (1.2 mL ± 0.2), the change in the volume of ACD 8% (v/v) solution (1.2 ± 0.2) mL, the change in the reaction time 20 ± 5 min and the change in the heating temperature 25 ± 5°C. These minor changes that may take place during the experimental operation didn’t affect the absorbance of the reactions product.

**Selectivity.** The selectivity of the methods was investigated by observing any interference encountered from the common tablet excipients such as starch, lactose, magnesium stearate and avisil. These excipients did not interfere with the proposed method.

**Specificity.** Other coformulated drugs specially amine containing drugs such as isopropamide, chlorphenramine maleate, paracetamol and caffeine did not interfere with the proposed method. Since these compounds are highly insoluble in chloroform and so easily eliminated without any interference.

## DISCUSSION

### Pharmaceutical Applications

The proposed method was successfully applied to determine the studied drug in its dosage forms. Tablet excipients such as starch, talc, lactose, magnesium stearate and avisil did not interfere with the proposed method. Statistically analysis of the results obtained and compared to those of a comparison method (35) using Student's *t*-test and the variance ratio F-test revealed no significant difference in the performance of the methods regarding the accuracy and precision respectively (Table 5).

**Table 5:** Application of the proposed and comparison methods for the determination of PPA in different dosage forms

Pharmaceutical preparation	Method I		Comparison method (35)
	Conc. taken (g/mL)	% Found	

Flustop^a^ tablets	20.00	99.1	99.77
	40.00	98.87	99.36
	60.00	99.7	100.6
X ± S.D.	99.22 ± 0.43		99.91 ± 0.63
t	0.69 (2.78)^d^		
F	2.14 (19.00)^d^		
Contac 12^b^ capsule	20.00	98.47	99.77
	40.00	98.87	99.36
	60.00	99.55	100.6
X ± S.D.	98.96 ± 0.55		99.91 ± 0.63
T	1.97 (2.78)^d^		
F	1.31 (19.00)^d^		
Pararhinol^c^ syrup	20.00	100.45	99.77
	40.00	100	99.36
	60.00	99.83	100.6
X ± S.D.	100.09 ± 0.32		99.91 ± 0.63
t	0.44 (2.78)^d^		
F	3.88 (19.00)^d^		

Each result is the average of three separate determinations.

a product of Glaxo Smithkline S.A.E Elsalam city, Cairo, A.R.E. each tablet labeled to contain 24mg PPA, 3mg chlorpheniramine maleate, 400 mg paracetamol and 32mg caffeine. batch# 043663A;

b product of EPICO. (10^th^ of Ramadan Egypt). batch#061682, each capsule labeled to contain 50mg PPA, 4mg of isopropamide;

c product of Misr CO for pharm.ind S.A.E Batch# 431046, each 5 mL labeled to contain 6.25mg PPA, 1mg chlorpheniramine maleate and 150mg paracetamol;

d Values between brackets are the tabulated t and F values, at *p*=0.05 (34).

### Mechanism of the reaction

**Figure 6:**
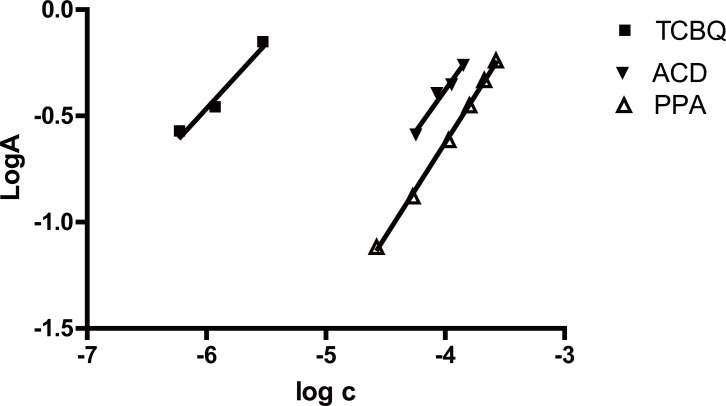
Limiting logarithmic plots for the molar reactivity of PPA with the proposed reagents, both of ACD and TCBQ respectively.

**Figure 7:**
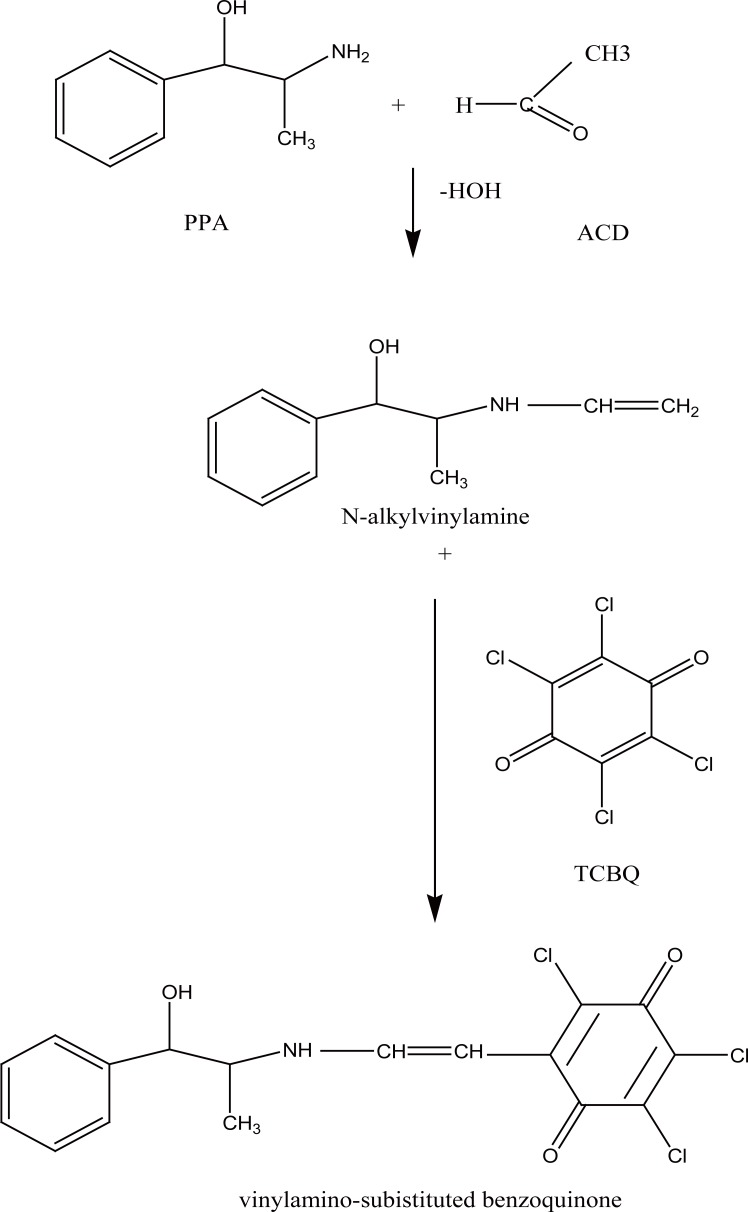
Proposed reaction pathway between TCBQ and PPA under the described reaction conditions.

The stoichiometry of the reaction was studied adopting the limiting logarithmic method in presence of excess of drug and reagent (36). The two straight lines were obtained using increasing concentrations of the two reagents while keeping the concentration of the drug constant and using increasing concentrations of the drug while keeping the concentration of the reagent constant. Plots of log absorbance *versus* log [TCBQ] and log [PPA] gave two straight lines with slope of 0.61/0.88 for TCBQ and PPA respectively, and 0.7872/0.8811 for ACD and PPA respectively (Fig. 6). Hence, it is concluded that the reaction proceeds in the ratio of 1:1 (TCBQ/PPA). Based on the obtained molar ratio and by analogy to previous study (32) it is clear that one molecule of the drug reacts with one molecule of ACD and one molecule of TCBQ. The proposed mechanism of the reaction is postulated to proceed as in the following scheme (Fig. 7).

## CONCLUSION

The proposed spectrophotometric method presents selective and simple, specific and inexpensive analytical procedures for determination of PPA, either *per se* or in its tablet dosage forms without interference from common excipients. Moreover, the developed method is time saving and do not require elaborate treatments associated with chromatographic methods. These attributes, in addition to the satisfactory sensitivity and reproducibility as well as the convenience and simplicity, make the proposed method suitable for routine analysis in quality control laboratories. Other coformulated drugs such as isopropamide, chlorphenramine maleate, paracetamol and caffeine did not interfere with the proposed method.
